# Co-Circulation of West Nile, Usutu, and Tick-Borne Encephalitis Viruses in the Same Area: A Great Challenge for Diagnostic and Blood and Organ Safety

**DOI:** 10.3390/v15020366

**Published:** 2023-01-27

**Authors:** Viktória Čabanová, Jana Kerlik, Peter Kirschner, Jana Rosochová, Boris Klempa, Monika Sláviková, Martina Ličková

**Affiliations:** 1 Institute of Virology, Biomedical Research Center, Slovak Academy of Sciences, Dúbravská Cesta 9, 845 05 Bratislava, Slovakia; 2 Regional Authority of Public Health in Banská Bystrica, Cesta k Nemocnici 1, 975 56 Banská Bystrica, Slovakia; 3 The General Hospital and Policlinic Lučenec n.o., Námestie Republiky 15, 984 39 Lučenec, Slovakia; 4 National Transfusion Service of the Slovak Republic, Ďumbierska 3/L, 831 01 Bratislava, Slovakia; 5 Department of Microbiology and Virology, Faculty of Natural Sciences, Comenius University in Bratislava, 84215 Bratislava, Slovakia

**Keywords:** flavivirus, co-circulation, emergence, neuroinvasive diseases, mosquito, tick

## Abstract

Viral infections caused by viruses from the family *Flaviviridae* such as Zika (ZIKV), Dengue (DENV), yellow fever (YFV), tick-borne encephalitis (TBEV), West Nile (WNV), and Usutu (USUV) are some of the most challenging diseases for recognition in clinical diagnostics and epidemiological tracking thanks to their short viremia, non-specific symptoms, and high cross-reactivity observed in laboratory techniques. In Central Europe, the most relevant endemic flaviviruses are mosquito-borne WNV and USUV, and tick-borne TBEV. All three viruses have been recognised to be responsible for human neuroinvasive diseases. Moreover, they are interrupting the blood and transplantation safety processes, when the great efforts made to save a patient’s life could be defeated by acquired infection from donors. Due to the trend of changing distribution and abundance of flaviviruses and their vectors influenced by global change, the co-circulation of WNV, USUV, and TBEV can be observed in the same area. In this perspective, we discuss the problems of flavivirus diagnostics and epidemiology monitoring in Slovakia as a model area of Central Europe, where co-circulation of WNV, USUV, and TBEV in the same zone has been recently detected. This new situation presents multiple challenges not only for diagnostics or surveillance but particularly also for blood and organ safety. We conclude that the current routinely used laboratory diagnostics and donor screening applied by the European Union (EU) regulations are out of date and the novel methods which have become available in recent years, e.g., next-gene sequencing or urine screening should be implemented immediately.

## 1. The Changing World of Flaviviruses and Their Vectors

Climate change, massive urbanization, rising transport, and travelling are responsible for changes in the distribution, spreading, and seasonality of flaviviruses and their vectors [1]. In Central Europe, three arboviruses from the family *Flaviviridae*, tick-borne encephalitis virus (TBEV), West Nile virus (WNV), and Usutu virus (USUV), are the main concerns for public health. The new era of global change has also brought significant changes to their distribution [1,2].

In the case of TBEV, the major vector of the European variant TBEV-Eu, the tick species *Ixodes ricinus* was found to occupy higher altitudes and a denser abundance was observed in the areas of its origin [3]. Additional competent vectors of TBEV, *Dermacentor reticulatus* and *Dermacentor marginatus* as tick species that are characteristic mainly of southern Europe, became more prevalent in Central Europe during the past decade [3].

A very similar behaviour was observed for vectors of WNV and USUV in Europe, mosquito species *Culex pipiens* s.l. and *Culex modestus*. *Cx. modestus* is considered principal bridge vector of WNV from reservoirs to dead-end hosts including humans. In the last decade, thermophilic *Cx. modestus* was found to be expanding its territory to the north thanks to global warming. The species was reported in several European countries for the first time, e.g., Denmark and Sweden [4,5]. *Cx. pipiens* s.l. is the main vector of WNV and USUV in Europe. This cryptic group consists of morphologically unidentified biotypes and species with different host preferences. Previously, it was considered that the biotype *pipiens* feeds dominantly on birds while the *molestus* biotype prefers the human host. Therefore, the transmission between bird reservoirs and humans is not particularly frequent. However, the hybridization between those biotypes has been observed in several parts of the world. Hybrids seem to not have strict host preferences, making them a great bridge vector for WNV and USUV. Experts believe that hybrids are responsible for epidemics in urban areas [6].

The introduction of invasive vectors into Europe also plays a very important role in the changing flaviviral epidemiology. Increasing travel and transport are responsible for introducing non-native species to new countries and their characteristic fast-adaptation results in their establishment and further spreading [7]. Besides, an invasive species also creates a huge potential for the introduction of non-native pathogens to new countries. *Aedes* invasive mosquitoes (AIM), the competent vectors of DENV, chikungunya (CHIKV), WNV, or even ZIKV, are a great example of such a pattern. In Europe, numerous outbreaks of re-emerging mosquito-borne diseases occurred after the establishment of the AIM species in the past decades [8]. For example, the outbreak of CHIKV with a total of 436 human cases appeared in Italy in 2007, while the only competent vector for this arbovirus in the area is an invasive tiger mosquito, *Aedes albopictus* [9]. A very similar scenario was observed with the alien tick *Rhipicephalus sanguineus* s.l., which prefers a warmer climate [10]. Global warming has prepared the environment for the spread and establishment of the tick in new countries. Moreover, with rising temperatures, this tick is more aggressive and linked to the human host [11,12]. *R. sanguineus* s.l. is the potential vector of the Crimean-Congo haemorrhagic fever virus (CCHFV) and other bacterial pathogens [13]. In addition, ongoing global warming shifts not only the vector distribution but also the prolongation of vectors’ and viruses’ seasonal activity. For example, the mild winters cause prolongation of the activity of ticks which leads to an increasing number of infected humans and changes in TBEV seasonality [14]. Extensive urbanization and destruction of the natural habitats of wild animals in the context of the extension of human settlements leads to another very important factor in the spreading of zoonotic diseases. The natural cycle between vectors and reservoir hosts is interrupted by humans which are more often in contact with wildlife and their diseases. For example, the TBEV surveillance study in ticks in Poland exposed a higher prevalence of TBEV in the city of Warsaw compared to more rural areas of the country [15].

## 2. Superior Criteria for Public Health Departments, Transplantation, and Blood Establishments in the European Union

In the European Union (EU) member states, several regulations and criteria have been applied for the diagnostics, donor screening, and epidemiology of WNV and TBEV.

For the public health departments, the Commission Implementing Decision (EU) 2018/945 on the communicable diseases are implemented [16]. The required laboratory criteria for WNV and TBEV case confirmation needs to be followed and at least one of the features has to be fulfilled (Table 1).

Directive 2002/98/EC setting standards of quality and safety for the collection, testing, processing, storage, and distribution of human blood and blood components is followed by blood establishments of the member states [17]. Regarding the viruses, the required criteria for regular blood donor screening are testing of Hepatitis B (HBV), Hepatitis C (HCV), and human immunodeficiency virus (HIV). However, the temporal criteria for donors are applied in connection to WNV. Restrictions and/or sample testing by a nucleic acid test (NAT) are implemented for donors residing or visiting WNV-affected areas (an affected area is an area with one and more locally acquired human cases) according to Directive 2004/33/EC and Directive 2014/110/EU [18,19].

To ensure human organs and tissue safety, Directive 2010/45/EU requires testing of HBV, HCV, and HIV [20].

Nowadays, no EU regulations refer to USUV transmission.

## 3. Overlapping Area of Co-Circulating WNV, USUV, and TBEV

In this perspective, we present the model situation from Slovakia, Central Europe, where a trend of the increasing flaviviral infections, with the changing vector distribution and the impact of global change led to the co-circulation of the three human pathogenic flaviviruses in the same area.

**Figure 1:**
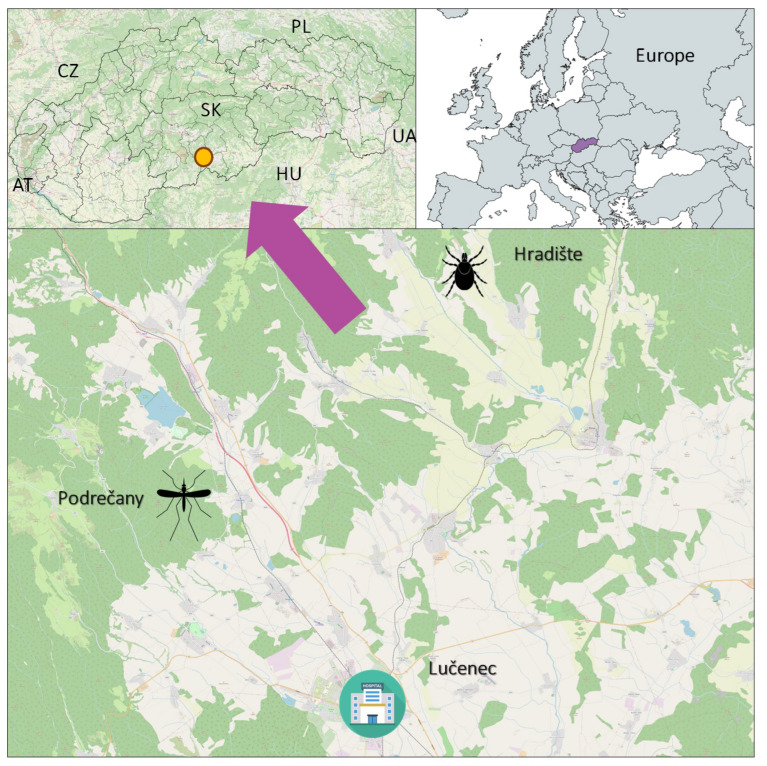
The area of co-circulation of West Nile (WNV), Usutu (USUV), and tick-borne encephalitis (TBEV) viruses in Slovakia, Central Europe. Legend: mosquito icon—WNV, USUV focus, tick icon—TBEV focus, hospital icon—the catchment hospital, orange dot—placement of the area in Slovakia, AT—Austria, CZ—Czechia, HU—Hungary, PL—Poland, SK—Slovakia, UA—Ukraine.

In the years 2018 and 2019, the molecular screening of selected flaviviruses in vectors revealed a co-circulation of WNV, USUV, and TBEV causative agents of neuroinvasive diseases in one area of the southern part of central Slovakia (Figure 1). RNA of WNV and USUV viruses was detected in their competent vector *Cx. pipiens* s.l. in the village Podrečany with a minimum infection rate (MIR) 3.10 [21]. RNA of TBEV was identified in the blood-fed tick, *I. ricinus*, harvested from a sheep on the farm in the village Hradište (Figure 1) [22].

**Figure 2:**
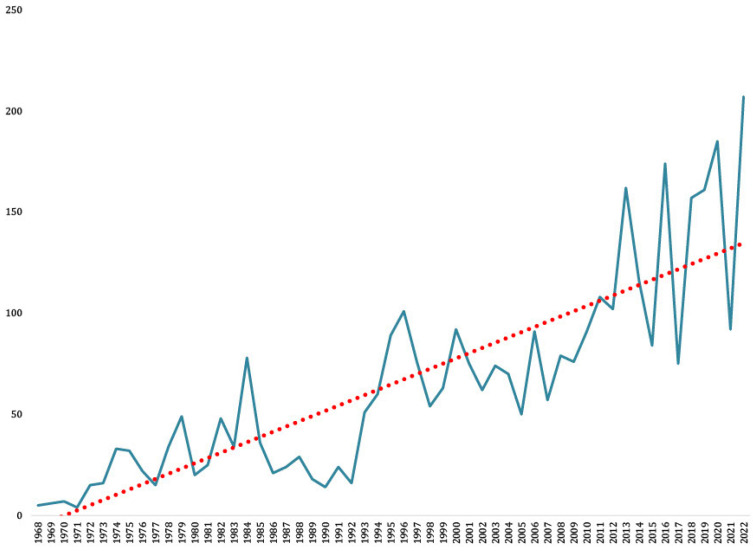
The total number of reported human cases of TBEV in Slovakia. Legend: the X axis represents years, the Y axis the number of cases. The red line represents a linear trendline.

From the area, only TBEV human cases were recognized [23]. TBEV is endemic in Slovakia with increasing incidence over the past 50 years (Figure 2) [23]. The increasing number of human cases in recent years is likely induced by several factors, e.g., behavioral changes as well as climate change [24]. Approximately 1% of all TBEV infections in Europe are caused by the alimentary route. This route of transmission typically leads to local outbreaks [25]. However, in Slovakia, up to 16–17% of all TBEV infections were through raw milk consumption [26,27]. During 2022, a total of 207 cases of TBEV with an incidence of 3.8/100,000 were reported from Slovakia according to data that were obtained from the Epidemiological Information System [23].

In contrast to the long history of TBEV human cases, the first human autochthonous case of West Nile fever (WNF) was diagnosed in southwestern Slovakia only in 2019. Thereafter, one neuroinvasive case was reported from the borderline area between Slovakia and Hungary, again in southwestern Slovakia in the summer of 2022. No human case of USUV has been detected in the country yet [21,23].

None of the viruses, TBEV, WNV, nor USUV, are regularly tested among the blood and organ donors in Slovakia, following the EU member state criteria. Only in the case WNV, screening is provided in an affected area when some human cases appear. In the studied area (Figure 1), where co-circulation of all three viruses was identified, no blood-donor testing for TBEV, WNV, and USUV has ever been performed.

Despite this, the lack of WNV and USUV human cases seems to be just a consequence of insufficient diagnostics and surveillance system in the country. Diagnostics of TBEV and WNV human cases have been monitored by the Public Health Authority of the Slovak Republic (PHA SR) only in suspected cases. USUV is not included in the diagnostics and/or monitoring in Slovakia yet.

Our hypothesis is also held up by our finding of a higher number of infected mosquitoes in the mentioned zone than in an already recognized WNV affected area in Slovakia. We found out that MIR of WNV in mosquitoes caught in Lučenec surrounding (Figure 1) was three-fold higher than MIR in mosquitoes collected in the Bratislava region, where two symptomatic WNV cases were diagnosed previously [21,23]. Furthermore, WNV has been diagnosed in a bird’s carcass in the neighboring district of Lučenec, Rimavská Sobota and TBEV antibodies were detected by the enzyme-linked immunosorbent assay (ELISA) and a plaque reduction neutralization test (PRNT) in sheep and goats in the farm Hradište (Figure 1) [22,28]. For this reason, it is highly probable the WNV and USUV cases might be overlooked and/or misdiagnosed as TBEV infections. There is a single hospital in Lučenec that presents the catchment hospital for several regions in this area (Figure 1). In the hospital, approximately 120 cases of aseptic encephalitis and meningitis are diagnosed per year while TBEV infections represent only a fraction of them [23]. In some of them, the causative agents remain unknown and those cases are not notifiable by PHA SR.

## 4. Nascent Challenges for Clinical and Laboratory Diagnostics

According to Kennedy et al. [29], up to 60% of viral encephalitis cases remain unidentified. The reason lies in the huge variety of possible causative agents, such as herpesviruses, paramyxoviruses, orthomyxoviruses, enteroviruses, retroviruses, alphaviruses, bunyaviruses, rhabdoviruses, parvoviruses, astroviruses, and finally flaviviruses. In the area of missing epidemiological data, the infectologists have no reason to suspect viruses such WNV and USUV to be responsible for neuroinvasive cases. In connection, the standard laboratory techniques are not helping to untie already complicated clinical diagnostics of viral neuroinvasive cases. The incubation period of flaviviruses ranges between 3 to 15 days post-infection. During this time range, the virus-generated viremia and RNA could be detected in the serum, plasma and cerebrospinal fluid (CSF) [30]. The RT-qPCR is one of the two diagnostic approaches and this method usually failed on the short period of viral RNA detectable in blood or CSF. For longer periods, WNV, USUV, and TBEV and also other viruses (e.g., ZIKV) are retained in the kidneys and are shed in the urine [31,32]. A more comprehensive clinical study proved that WNV infection in neuroinvasive cases can be more reliably and effectively diagnosed from urine samples than from CSF [31,32]. Urine samples are also suitable biological material for virus isolation which can help for further, more detailed investigation of the causative agents. Unfortunately, urine samples do not seem to be taken as standard sampled material yet [31,33].

The second laboratory technique that is standardly used for flaviviruses is based on antibody detection. The immune system starts to create antibodies between 4 and 8 days after the beginning of viremia or the onset of symptoms. Immunoglobulin G and M antibodies against flaviviruses can persist in blood serum for several years [34]. However, even serology has its own limitations. Among the whole family *Flaviviridae*, a high cross-reactivity of antibodies is observed and the validation of the commonly-used ELISA method is required.

As the gold standard for ELISA confirmation, a plaque reduction virus-neutralization test (PRNT) is considered, even though the test requires maintenance in the BSL3 laboratories (for WNV and TBEV). Regrettably, once the diagnostics seems to be solved, the story becomes even more complicated. PRNT is not effective for the acute phase of infection (IgM), but eligible for persistent IgG antibodies that are usually generated at 14 days onset. Moreover, in the area of flavivirus co-circulation, PRNT needs to be performed against several flaviviruses (e.g., USUV, WNV, and TBEV) or because of the travel anamnesis, non-native flaviviruses need to be considered (e.g., YFV, DENV, ZIKV) [35].

Let us imagine the situation in our model area with co-circulation of WNV, USUV, and TBEV. It is probable the patient underwent several infections and retains antibodies against more than one flavivirus. Multiple favivirus antibodies will cause cross-reactivity on ELISA but will be also difficult to distinguish in PRNT. The same observation was already done during the WNV and USUV surveillance among the bird population in Germany. High titres of both viruses, e.g., WNV 1:960 and USUV 1:480, were simultaneously found in three sera by virus-neutralization tests. The authors suspect those cases as co-infections, because those individuals were captured in the same aviary. However, the exact interpretation of cross-reactivity and co-infection during virus-neutralization test is unclear [36].

A very similar situation is observed in countries with co-circulating ZIKV and DENV. The laboratory confirmation based on serology, therefore, does not appear to be reliable. Moreover, in the dual humoral response, we could observe protection, but also the virus-enhancing role of antibodies within the *Flaviviridae* family [37,38]. In the case of closely antigenically related viruses WNV and USUV, a chimeric vaccine constructed by WNV precursor membrane (prM) and envelope (E) proteins provides protection also against USUV in the mice models [39]. More complicated seems to be a relationship between WNV and TBEV antibodies. According to the older study by Phillpotts et al., prior TBEV antibodies can have an enhancing effect on WNV infectivity, but the opposite effect to TBEV enhancement by WNV antibodies was not observed [40]. Hypothetically, this scenario can cause problems for the TBEV vaccinated population in regions of WNV circulation. However, the antibody-dependent activity between WNV, USUV, and TBEV needs to be further studied.

In this moment, human vaccines against USUV and WNV are not available. However, active immunization is the most important protection against TBEV. All TBEV vaccines registered in EU are based on the inactivated whole virus, but due to several factors, the upgraded TBEV vaccine strategies were already requested by experts [41]. During the flavivirus vaccines development, antibody-dependent enhancement among the family *Flaviviridae* should be highly considered. Several epitopes of antibodies were already revealed to have an enhancement effect. Specifically, prM and the fusion loop of E antibodies were shown to have an enhancing role [38]. The non-structural protein 1 (NS1) appears to be a better candidate for flavivirus vaccines with no role in virus-enhancement [42].

Nowadays, we found Europe in the same situation as is noticed in the endemic areas of ZIKV and DENV, where antibodies testing should be considered very carefully, because cross-reactivity, co-circulation, prior infection, and vaccination can highly influence the obtained results [43]. It, therefore, seems that epidemiological data are also an essential tool even for diagnostics, but are still very limited in some European countries [44,45].

## 5. Are Blood Components and Transplanted Organs Still Safe?

Nowadays, in the era of an increasing number of WNV human cases, special attention has been given to the safety of blood, blood components, and transplanted organs, because several infections were reported via transfusion and transplantation from infected donors. Unfortunately, WNV clinical course in blood or organ recipients is much more complicated, with the majority of neuroinvasive forms and the number of fatal cases being 10-fold higher than in the general population. For this reason, EU countries apply measures to warrant blood safety towards WNV infections [46].

Currently, there is no testing of the blood or organ donors for the presence of TBEV. However, blood transfusion was reported as the mode of TBEV transmission by Wahlberg et al. [47]. The authors observed two cases of TBEV-infected blood donors in Finland, one male and one female with a biphasic course with symptoms only in the second phase. Lipowski et al. [48] reported organ transplantation as the mode of transmission of TBEV. This study presents the transmission of the virus through transplanted organs (liver and kidneys) from a single infected donor (male) to three recipients (males) in Poland. The recipients became ill 17–51 days after transplantation and all died 36–83 days later in the hospital. TBEV infection was confirmed in all four people (donor and three recipients) by necropsy, next-generation sequencing (NGs), and reverse transcriptase polymerase chain reaction (RT-PCR). Immunosuppressive therapy probably increases the risk for development of fatal courses of TBEV infection [48].

USUV is also frequent among blood donors in some countries such as Hungary and Austria and we already know USUV infections can lead to neuroinvasive diseases. Nonetheless, it is currently not considered to be included in routine donor testing [49,50].

If the blood or organ donor screening is performed, RNA is obtained mainly from the blood samples. As described above, the active virus can be found in the kidney for a much longer period, e.g., for WNV from 1 to 41 days after symptom onset [31]. This presents a great risk for acquired infection during kidney transplantation. According to the Global Observatory on Donation and Transplantation, more than 100,000 kidneys are transplanted annually and the kidney is the most transplanted organ [51]. If the urine samples are included in the routine donor screening too, first, the efficacy of the method will increase and second, the most vulnerable organ recipients will be protected [31].

As a part of the action to mitigate the risk to take blood from asymptomatic donors as part of an EU regulation superior for blood establishments of member states, restrictions and/or sample testing were implemented for donors residing or visiting WNV-affected area [52]. But at that point, the epidemiological data of flaviviruses circulation among human and vector populations needs to be available to implement such steps.

## 6. The Call for the Novelty to Protect Human Health

The trend of increasing flaviviral infections and changing distribution of viruses and vectors are the main pressure to change and update standard diagnostic procedures which are out of date. Novel approaches were already requested for the diagnostics of Zika and Dengue for very similar reasons as we already mentioned: (a) short-diagnostic window for acute infection, (b) extensive cross-reactivity in serology, and (c) missing tools for clinical diagnostics. RT-qPCR, IgM, and PRNT have been standard diagnostic methods for flavivirus infections for more than 25 years [53]. The current approaches in the routine diagnostics and donor screening are not sufficiently handling the onslaught of global change. Once again, we should highlight the importance and reason for this call. Flaviviruses are causative agents of severe and in some cases fatal human and animal diseases with immense public health importance.

For a few years already, several scientific publications have suggested the use of viral-targeted NGs in diagnostics [29,54]. Due to fast mutation of RNA viruses such as flaviviruses, targeted detection needs to be updated all the time. In contrast, untargeted RNA sequencing can help to generate new information without the loss of sensitivity for diagnostics purposes. However, the challenging aspect of rapid sequencing dwells in the relatively low abundance of viral RNA compared to host RNA. Nowadays, even those limitations can be solved by (a) an enrichment of viral RNA or (b) depletion of ribosomal RNA [55]. The results performed by virome sequencing from sampled biological material can reveal the causative agent for up to 72 h [29]. Moreover, the whole genome sequencing of neuroinvasive cases is helping to investigate the pattern leading to the development of more severe forms of diseases. In connection with genetic and virologic studies that were performed on currently circulating strains, preparedness and risk assessments can be implemented much faster and rapidly according to relevant data for an area. This approach has been a very successful tool in handling the current COVID-19 pandemic and shown to be feasible and to have direct impact also on a local scale, not only globally [56]. Nowadays, the cost of NGs is comparable or even lower than standard laboratory techniques which makes this method affordable and available as a routine laboratory diagnostic method [57]. Furthermore, we should finally consider non-native viruses, such as DENV, ZIKV, and CHIKV approaching as potential causative agents of viral infection and include them into the differential diagnostics [58].

## Figures and Tables

**Table 1:** Required laboratory criteria for the case confirmation according to the Commission Implementing Decision (EU) 2018/945 on communicable diseases. At least one of the following criteria needs to be fulfilled.

Laboratory Test	Isolation of Virus from Clinical Specimen	Detection of Virus’ Nucleic Acid in Clinical Specimen	Specific Antibody Response (IgM) in CSF	IgM High Titre + Detection of IgG + Confirmation by Neutralisation	Seroconversion or Four-Fold Increase of Specific Antibodies in Paired Serum Samples
WNV	✔	✔	✔	✔	✕
TBEV	✔	✔	✔	✔ *	✔

WNV—West Nile virus, TBEV—Tick-borne encephalitis, CSF—cerebrospinal fluid, Ig—immunoglobulin. * The confirmation by neutralisation and IgM high titre is not required for TBEV.

## Data Availability

Not applicable.
